# Microfluidic Assessment of Frying Oil Degradation

**DOI:** 10.1038/srep27970

**Published:** 2016-06-17

**Authors:** Mei Liu, Shaorong Xie, Ji Ge, Zhensong Xu, Zhizheng Wu, Changhai Ru, Jun Luo, Yu Sun

**Affiliations:** 1 Shanghai Key Laboratory of Intelligent Manufacturing and Robotics, School of Mechatronic Engineering and Automation, Shanghai University 200072 China; 2 State Key Laboratory of Transducer Technology, Shanghai Institute of Microsystem and Information Technology, Chinese Academy of Sciences, Shanghai 200050, China; 3 Department of Mechanical and Industrial Engineering, University of Toronto, Toronto, ON, M5S 3G8, Canada; 4 Jiangsu Provincial Key Laboratory of Advanced Robotics and Collaborative Innovation Center of Suzhou Nano Science and Technology, Soochow University, Suzhou, 215123, China

## Abstract

Monitoring the quality of frying oil is important for the health of consumers. This paper reports a microfluidic technique for rapidly quantifying the degradation of frying oil. The microfluidic device generates monodispersed water-in-oil droplets and exploits viscosity and interfacial tension changes of frying oil samples over their frying/degradation process. The measured parameters were correlated to the total polar material percentage that is widely used in the food industry. The results reveal that the steady-state length of droplets can be used for unambiguously assessing frying oil quality degradation.

Frying is one of the oldest and most popular cooking methods globally. Due to cost, frying oil is repeatedly used. The assessment of used frying oil is a subject of great concern for health agencies, food manufacturers, and consumers. The most frequent chemical reactions occurring in the frying process are hydrolytic reactions, oxidation reaction, and thermal alteration^1^. The generated thermoxidation compounds deserve attention as the oxidative stress is associated with various degenerative processes and diseases, for example, mutagenesis, cell transformation and cancer, atherosclerosis, heart attacks and chronic inflammatory diseases^2^.

For quality control, regulations over the discard time point of frying oil have been established in many countries, which define the limit of total polar materials (TPM), oxidised fatty acids (OXF), polymerised triglycerides (PTGs), free fat acid (FFA), foaming point, acid value, and carbonyl compounds^3^,^4^. The most widely accepted standard is that used frying oil must be replaced when its TPM exceeds 24%^5^,^6^,^7^.

Since it is time consuming and labor-intensive to measure TPM directly, related parameters are often used for assessing frying oil quality. Devices measuring the dielectric constant of frying oil are commercially available (e.g., CapSens 5000, Switzerland; FOM 310, Germany; and Testo 265, 270, Germany). In these devices correlation between dielectric constant and TPM percentage has been established^7^,^8^,^9^. Electric conductivity was also demonstrated as a measure; however, this measure has not been correlated with TPM, and instrument operation is time consuming^10^,^11^. As polymer compounds formed during frying lead to increased viscosity, viscosity has also been considered as an indicator of frying oil quality. The correlation between viscosity and TPM has been established^12^,^13^,^14^,^15^.

Near-infrared spectroscopy (NIRS) has been proven powerful for monitoring frying oil quality. It is capable of measuring a number of chemical parameters including TPM, PTG, FFA, peroxides, anisidine value, and carbonyl value^9^,^16^,^17^,^18^,^19^,^20^. Other techniques attempted for frying oil quality assessment include image processing^21^, E-nose^22^,^23^, pore-based wicking sensor^24^,^25^, HPTLC-densitometry^26^, and ultrasonic technique^27^,^28^. This work aimed at developing an easy-to-use, rapid, and disposable microfluidic device for assessing the quality of frying oil, based on their viscosities and oil/water interfacial tension changes. Although there exist microfluidic devices for viscosity measurement, they either are only suitable for low viscosity measurement (<60 cP)^29^,^30^,^31^ while the viscosity of frying oil is typically in the range of 50–150 cP at room temperature^13^,^14^,^32^, or suffer from wall slip and shear banding problems^33^,^34^. Our microfluidic device generates water-in-oil droplets and characterizes their size to quantify the degradation of frying oils. By comparing with TPM, the microfluidic device demonstrated its capability of quantitatively evaluating frying oil quality.

## System and principles

### Device design and principles

**Figure 1:**
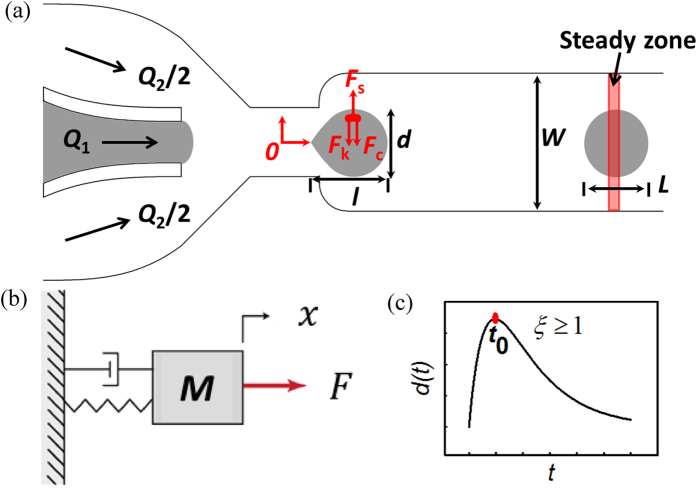
(**a**) Schematic of the microfluidic device for measuring frying oil quality. *Q*_1_ and *Q*_2_ are respectively the rate of the inner (deionized water) flow and sheath flow (frying oil). *W* is width of the microchannel, *L* is the steady-state length of the droplet, and *l* and *d* are the dynamic length and width of the droplet. (**b,c**) Mass-damper-spring vibration system for modeling droplet dynamics, and the impulse response of the mass-damper-spring system with *ξ* ≥ 1, where *x* is the displacement of droplet surface from the balance position, *ξ* is the damping ratio of the system, and *t*_0_ is the time instance when the droplet has the largest deformation.

Figure 1 shows a schematic of the microfluidic device. The device has two sheath flow inlets and one inner sample flow inlet. Frying oil (continuous phase) and deionized water (dispersed phase) are injected simultaneously into the microchannel as the sheath flow and inner flow, respectively, and water-in-oil droplets are generated at specific flow rates^33^. The dimension of the droplets in the microchannel depends on the channel size, oil-water flow rate ratio, and capillary number of the sheath oil flow. The normalized steady-state length of the droplet can be predicted according to


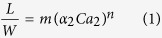



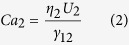
where *L* is the steady-state length of the droplet; *W* and *h* are the microchannel width and height; *m* and *n* are coefficients determined by microfluidic channel geometries, dimensions, and wettability. 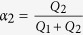 is the volume fraction of frying oil; *Ca*_2_ is the capillary number of the oil phase, characterizing the ratio of viscous forces to interfacial tension forces; 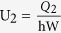 and *η*_2_ are the average flow velocity and viscosity of oil phase; and *γ*_12_ is the interfacial tension between frying oil and deionized water. A “steady zone” is defined to be sufficiently far down the microchannel, as labeled in Fig. 1(a). When a droplet enters the steady zone, it has fully developed and reached the steady state when the steady-state length *L* of the droplet is measured.

Eq. (1) can be rewritten as


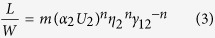


During the droplet generation process, in cases of no occlusion, as flow rates are fixed, strong viscous force favors droplet pinch-off and tends to reduce droplet size, and is negatively related with *L*/*W* while interfacial tension acts against droplet pinch-off and tends to enlarge the droplet, and is positively related with *L*/*W*. Therefore, the value of *n* is negative^35^, which was confirmed in our experiments (see Supplementary Figure 4). As discussed quantitatively in the next section, oil viscosity increases over the frying process, and in the meanwhile, oil/water interfacial tension decreases as a consequence of the formation of polar groups^36^,^37^. Thus, with flow rates kept constant, the capillary number *Ca*_2_ of the oil phase increases with frying (Equation (2)), leading to decreased *L* (note: *n* is negative). In summary, with *Q*_1_ and *Q*_2_ kept constant, heavier frying leads to decreased *L*. Based on this principle, oil degradation can possibly be distinguished and quantified.

### Characterization of droplet deformation

On the microfluidic device, due to the convergence followed by the widening of the nozzle structure, the generated droplet undergoes a shape deforming process. The deformation index (*DI*) of droplets is defined as *l*/*d*, where *l* and *d* are the dynamic length and width of the droplet, as shown in Fig. 1(a). A time zero point (“*O*” in Fig. 1) located at the center of the nozzle is defined, and *t*_*0*_, the time instance when the droplet widens the most, is also defined and explored as a parameter for distinguishing oil degradation differences since it is determined by and reflects oil viscosity and oil/water interfacial tension.

During the droplet deforming process, in cases of no occlusion, in the transverse direction, the droplet experiences a shear force (*F*_s_ in Fig. 1) generated by the widening microchannel, a viscous force (*F*_c_) and an interfacial tension force (*F*_k_). For simplicity, the transverse shear force can be described by an impulse function. As shown in Fig. 1(b), the droplet deforming dynamics is


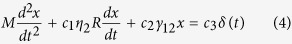


where *x* is the displacement of the droplet surface from the balance position; *M* is the mass of the droplet; *R* is the radius of a sphere of the same volume as the droplet; and c_1_, c_2_ and c_3_ are coefficients dependent on geometries of the droplet, the microchannel, and initial conditions. Since the water droplets in different oils have similar geometries, their c_1_, c_2_ and c_3_ values are approximately identical.

This model can be rewritten as


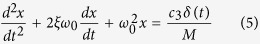


where the undamped natural frequency is 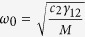, and damping ratio 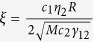. Since no oscillation is observed in experiments, *ξ* **≥ **1 and the impulse response of the droplet is written as


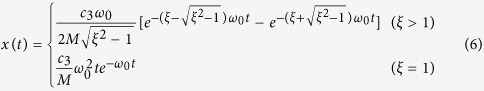


The droplet dynamic width *d* is therefore


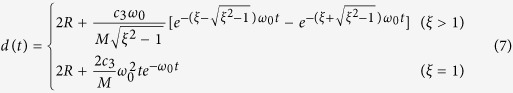


As shown in Fig. 1(c), the droplet dynamic width *d* reaches the maximal value and then decreases to a constant value. On the contrary, as the droplet area remains constant, the droplet dynamic length *l* decreases first then increases to a constant value, resulting in a decreasing and then increasing *DI*. Eventually (i.e., in steady state) *DI* also reaches a constant value. The time instance when the droplet widens the most, *t*_*0*_, can be derived from Eq. (7) and is


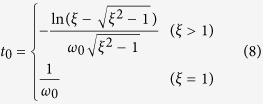


Obviously, if *ξ* ≠ 1,* ξ* and *ω*_0_ both affect *t*_0_. In our case (*ξ* ≥ 1), *t*_0_ is more dominated by *ω*_0_, which means *t*_0_ is largely dependent on *M* and *γ*_12_. This can be attributed to the fact that at this scale interfacial tension is dominant over viscous force (0.01 < Ca < 0.04). At the same flow rate, for droplets in more heavily fried oil, droplet mass *M* and interfacial tension γ_12_ both decrease. Thus, *t*_0_ can be a potentially useful parameter for distinguishing fry oils with different degradations, if the change of *M* is negligible compared to that of *γ*_12_.

## Materials and Methods

Canola oil samples (Mazola, ACH Food Companies, Inc.) were used in this study. Initially, 1,200 mL oil was heated up for 1 h at 180–185 °C. For each 20-min frying cycle, 200 g of chicken nuggets were fried. Twelve frying cycles were performed every day, for two consecutive days without any oil replenishment. A 150 mL sample was collected after every six frying cycles. Prior to every sample collection, oil was agitated to achieve a complete mixing to obtain a homogenous representative sample. Thus, four frying oil samples were obtained and denoted as FO1-FO4 while the original oil was denoted as FO0. All samples were filtered to remove suspended food particles for measurements.

Detailed physical properties of the samples were measured. Viscosity was measured using a DV-III Ultra Programmable rheometer (Brookfield, Massachusetts). The interfacial tension between the water droplet phase and oil phase was measured using the Du-Nouy ring method (Sigma700, KSV Instruments LTD). TPM of the oil samples was measured using a commercially available device (Testo 270, Testo Inc., Germany).

The microfluidic devices were constructed with PDMS using standard soft lithography. The width of the main channel (i.e., *W* in Fig. 1) is 1,000 μm. The height of all microfluidic structures is 80 μm. All microfluidic experiments were conducted at room temperature of 23 °C. An oil sample was symmetrically loaded into the sheath flow inlet at a flow rate of 0.6 mL/h by a syringe pump, and deionized water was loaded into the inner flow inlet at flow rates ranging from 0.05 mL/h to 0.25 mL/h at a step of 0.05 mL/h by a second syringe pump. The flow rates were experimentally selected by taking into account the maintenance of device integrity and the uniformity in droplet generation. No cases of occlusion were included in this study.

Droplet flowing videos were captured at a speed of 500 frames·s^−1^ (HiSpec 1, Fastec Imaging Corp., U.S.), under a 4× objective of an inverted microscope (3.45 μm/pixel). A custom-developed MATLAB image processing program was used for automated droplet image analysis. When a droplet reaches the zero point (“*O*” in Fig. 1), droplet length and width start to be measured and are continuously monitored until the droplet leaves the ‘steady zone’ to quantify their dynamic dimensional characteristics.

## Results and Discussion

### Physical properties of the tested oil samples

**Figure 2:**
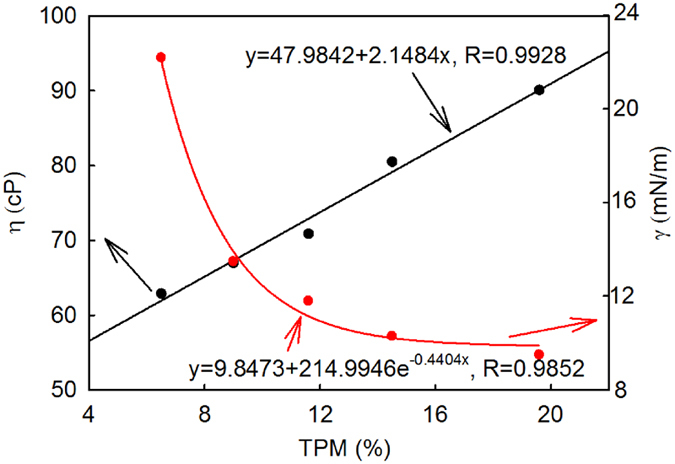
Experimentally measured TPM vs. experimentally measured interfacial tension and viscosity values of the tested oil samples.

Table 1 summarizes the physical properties of the oil samples used in this study. It can be seen that over the frying process, oil viscosity and TPM consistently increased while the interfacial tension consistently decreased. Oil density after the 24 frying cycles (each cycle: 20 minutes) remained approximately constant. Figure 2 shows that the TPM values of the five oil samples linearly correlate with viscosity and exponentially correlate with interfacial tension, in agreement with previously reported results^2^,^9^,^26^,^27^,^28^.

### Microfluidic assessment of frying oil degradation

**Figure 3:**
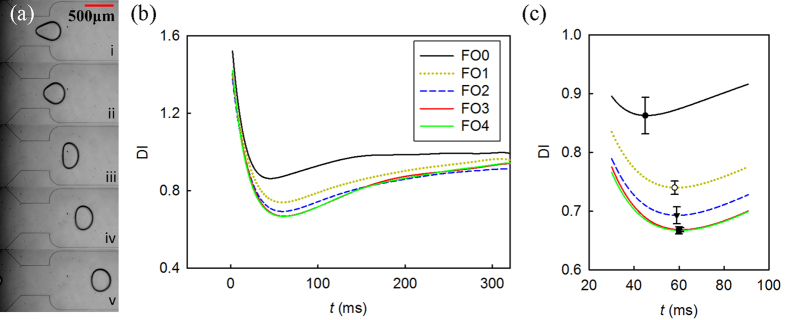
(**a**) Droplet deforming process. (**b**) Droplet deformation index vs. time. *Q*_1_ = 0.05 mL/h, *Q*_2_ = 1.2 mL/h. (**c**) Zoom-in of the trough areas in (**b**). Time zero is the time instance when the rear of the droplet reaches zero point labeled in Fig. 1. Error bars indicate the positions of *t*_0_.

We first investigated the use of the deformation index, *DI* = *l*/*d*, for assessing frying oil degradation. On the microfluidic device, once the rear of a droplet reached the zero point, the dimensions of the droplet started to be measured and continued to be monitored until 300 ms when droplets reached the steady state (see Supplementary Video). As shown in Fig. 3(a), when the droplet exits the nozzle, it reveals a widening and recovering behavior, as predicted by Eq. (7) and Fig. 1(c). The as-generated droplet has a spindle-like shape [Fig. 3(a)i]. It was then stretched transversely due to shear stress [Fig. 3(a)ii–iii], and its *DI* value rapidly decreased. In the recovering process [Fig. 3(a)iii–v], viscous force and interfacial tension work together to minimize viscous resistance and interfacial energy, causing the droplet to become more spherical.

For all the water-in-oil droplets in this study, the interfacial force was much stronger than the viscous force (0.01 < Ca < 0.04). Consequently, the interfacial resistance was stronger for droplets encapsulated with less used frying oil which has higher interfacial tension (see Table 1). Therefore, droplets encapsulated with less used frying oil widened more slowly. The *ω*_0_ value of the droplets generated with the five oil samples were estimated to be 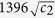, 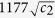, 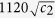, 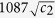, 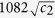, respectively with Q_1_ = 0.05 mL/h and Q_2_ = 1.2 mL/h. Since *t*_0_ is inversely dependent on *ω*_0_, water droplets in less used oil result in shorter *t*_0_, as shown in Fig. 3(b,c). On the other hand, due to inevitable changes in *M* resulting from changes in droplet size, droplets generated with FO3 and FO4 have very close *ω*_0_, which explains the almost identical *t*_0_ values for oil samples FO3 and FO4 as shown in Fig. 3(c). It can be seen that *t*_0_ is effective for distinguishing FO0, FO1, and FO2; however, because of their almost identical *t*_0_ values, FO3 and FO4 cannot be effectively distinguished.

**Figure 4:**
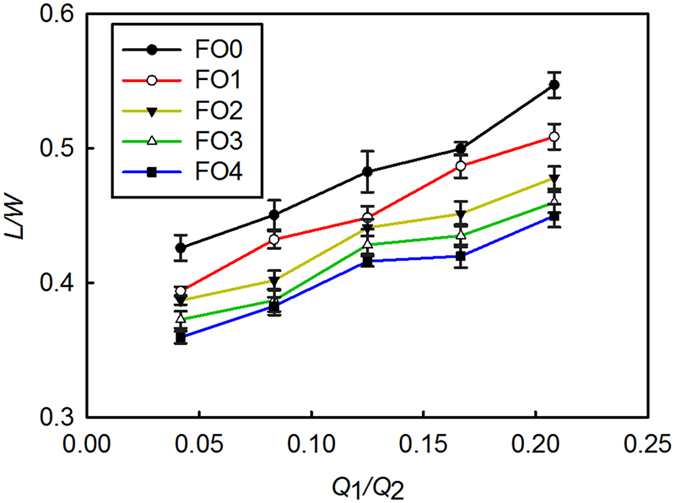
Normalized steady-state length of the droplets vs. different flow rate ratios, Q_1_/Q_2_. Q_2_ was set at 1.2 mL/h. n > 32 for each data point.

We then explored the relationship between droplets’ steady-state length, *L* and oil degradation. Figure 4 shows experimental data measured at different flow rates. Although Eq. (1) is for cross-shaped microchannels, and the focusing structure on our microfluidic device is a converging nozzle, the model still properly predicts the trend of flow behavior (i.e., as α_2_Ca_2_ increases, *L*/*W* decreases; data not shown). As revealed by Eq. (1), increase in the flow rate ratio and oil viscosity or decrease in interfacial tension can result in a shorter steady-state length of droplets. This is confirmed by experimental data summarized in Fig. 4 which shows that when *Q*_1_/*Q*_2_ was increased, *L*/*W* of water-in-oil droplets also increased. The data curves of oil samples FO0-FO4 distinctly separate themselves from each other, due to the fundamental differences in their viscosity and interfacial tension. After the original oil was used for more frying cycles, viscosity became higher and interfacial tension became lower (see Table 1). This combined effect caused the steady-state length, *L* to become shorter for used frying oil. As can be seen in Fig. 4, for a given ratio of *Q*_1_/*Q*_2_, the steady-state length of the droplets consistently decreased over the frying period (i.e., from original oil FO0 to most used FO4). The variations shown in Fig. 4 from multiple measurements were mainly from pulsating flow of the mechanical pumps and image processing for measuring droplet parameters.

**Figure 5:**
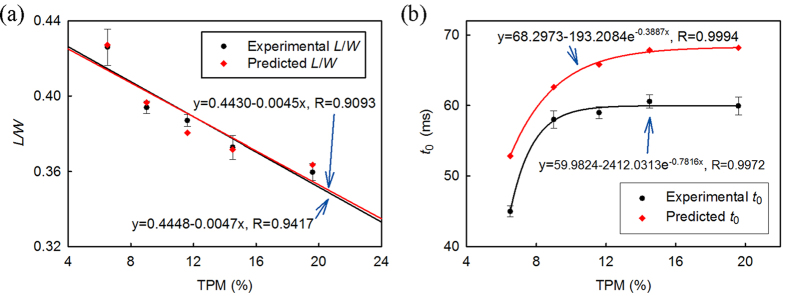
(**a**) Correlation between theoretical and experimentally measured *L*/*W* and experimentally measured TPM. The relationship is highly linear and proves that *L*/*W* is a suitable parameter for discriminating oil samples FO0-FO4. *L*/*W* reaches 332 μm (by experiment)/335 μm (by prediction) when TPM reaches 24%. (**b**) Correlation between theoretical and experimentally measured *t*_0_ and experimentally measured TPM. Data presented in this figure were collected with Q_1_ = 0.05 mL/h, Q_2_ = 1.2 mL/h.

Finally, we correlated experimentally measured microfluidic parameters with the oil samples’ TPM values. The experimental and theoretical results shown in Fig. 5 confirm that normalized steady-state length (*L/W*) linearly correlates to TPM and *t*_0_ exponentially increases vs. TPM. The difference between model predictions, based on Eq. (1) and Eq. (8), and experimental measurements is <2% for *L/W* and <17% for *t*_0_. Based on the measured data, the sensitivity of the system was determined to be 0.0047/TPM, and its precision is ±1.14% TPM (vs. ±2% TPM for Testo 270). The highly linear relationship between the steady-state length and TPM also indicates that the oil should be replaced when the steady-state length of the droplets drops below ~335 μm, corresponding TPM of 24%, although oil samples with TPM exceeding 24% were not tested in this study.

As seen in Fig. 5, with a decreasing *M*, which results from droplet size reduction, *t*_0_ increases to a constant value rapidly. Although FO3 and FO4 have large differences in their TPM values (14.5% vs. 19.6%), their generated droplets had almost identical *t*_0_ values that are different by only 3%. In contrast, consistently with data presented in Fig. 4, *L*/*W* is a more suitable parameter than *t*_0_ for assessing FO0-FO4 in terms of both linear correlation to TPM and clear discrimination of all the tested samples. These results represent the first quantitative correlation between the microfluidic parameter *L/W* and oil TPM and imply that *L/W* could be a suitable metric for conveniently assessing frying oil degradation. Future field deployment of this technology would benefit from advances in the development of miniaturized peripheral instruments such as micro pumps^38^,^39^,^40^ and imaging tools^41^,^42^,^43^,^44^.

## Conclusion

In this work, frying oil degradation over the frying process was quantitatively assessed, using a microfluidic approach. Mono-dispersed water-in-oil droplets were generated by a hydrodynamic focusing microfluidic device. Measurements were made by high-speed imaging and automated image processing. The results proved that as frying time increased, oil viscosity increased and oil/water interfacial tension decreased. Consequently, the steady length of the generated water-in-oil droplets decreased over the frying process. By correlating droplet steady-state length to TPM, the tested frying oil samples were distinctly distinguished and quantitatively separated. The results indicate that droplet stead-state length can be a suitable parameter for conveniently quantifying frying oil degradation.

## Additional Information

**How to cite this article**: Liu, M. *et al.* Microfluidic Assessment of Frying Oil Degradation. *Sci. Rep.* **6**, 27970; doi: 10.1038/srep27970 (2016).

## Supplementary Material

Supplementary Information

Supplementary Movie

Supplementary Information

## Figures and Tables

**Table 1:** Physical properties of the oil samples studied in this work.

Oil sample	Density ρ (g/mL) 22 °C	Viscosity η (mPa·s) 24.1 ± 0.1 °C	Interfacial tension γ (mN/m) 22.7 ± 0.3 °C	TPM (%) 43.0 ± 0.4 °C
FO0	0.93 ± 0.01	62.9 ± 0.4	22.2 ± 0.3	6.5 ± 0
FO1	0.94 ± 0.02	67.0 ± 0.6	13.5 ± 0.2	9 ± 0
FO2	0.94 ± 0.01	70.9 ± 0.9	11.8 ± 0.3	11.6 ± 0.2
FO3	0.94 ± 0.01	80.5 ± 0.3	10.3 ± 0.3	14.5 ± 0
FO4	0.95 ± 0.01	90.1 ± 0.4	9.5 ± 0.2	19.6 ± 0.2
