# Supplementary material for: Microfluidic Assessment of Frying Oil Degradation
Source: Sci Rep. 2016 Jun 17;6:27970. doi: 10.1038/srep27970 (PMC4911549; doi:10.1038/srep27970)
Supplement: Supplementary Information [file srep27970-s1.pdf]

Microfluidic Assessment of Frying Oil Degradation

Mei Liu[1,2,3], Shaorong Xie[1,3*], Ji Ge[1, 3], Zhensong Xu[3], Zhizheng Wu[1], Changhai Ru[1,4], Jun Luo[1,3*], and Yu Sun[1,3*]

Supplementary Information

1. Droplet deforming behavior under another set of experimental condition

Supplement Fig. 1 shows that, when Q_1=0.2mL/h and Q_2=1.2mL/h, as the droplet exits the nozzle, it undergoes a shape deforming process: *DI* decrease first then increase to a constant, which means that the droplet stretches first then shrinks in the transverse direction.

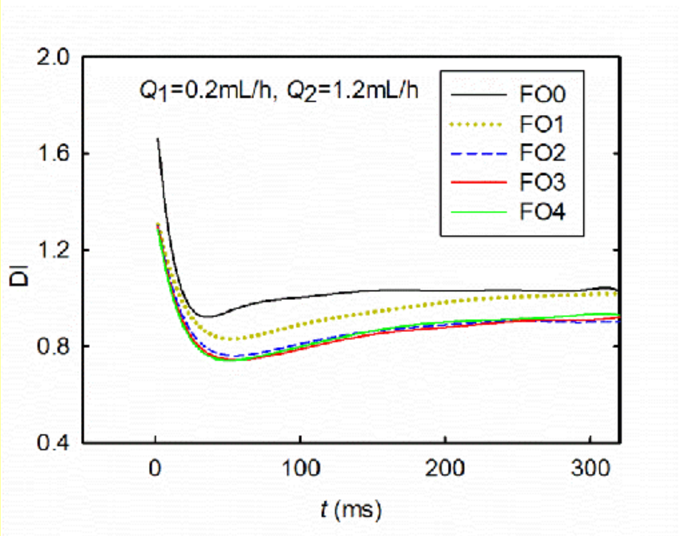

Supplement Fig. 1: Droplet deformation index vs. time. Q_1=0.2mL/h, Q_2=1.2mL/h. W=1000μm, h=80 μm.

2. Results from another set of experiment

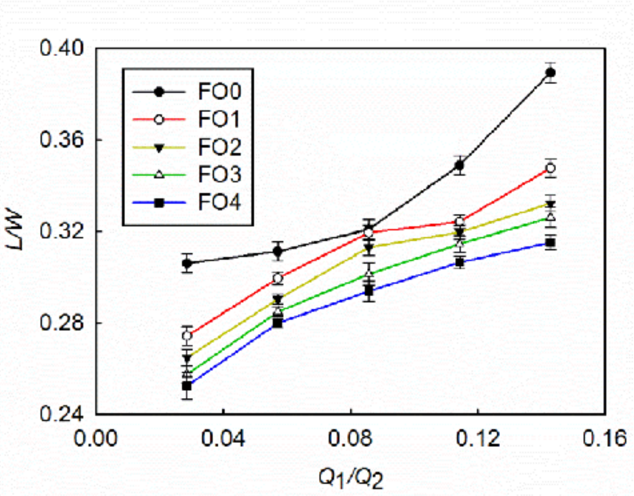

Supplement Fig. 2: Normalized steady-state length of the droplets vs. different flow rate ratios, Q_1/Q_2. Q_2 was set at 0.7mL/h. W=1000μm, h=40 μm. n>38 for each data point.

Supplement Fig. 2 shows that, when Q_1/Q_2 was increased, L/W of water-in-oil droplets also increased. The data curves of oil samples FO0-FO4 distinctly separate themselves from each other, due to the fundamental differences in their viscosity and interfacial tension. For a given ratio of Q_1/Q_2, the steady-state length of the droplets

consistently decreased over the frying period.

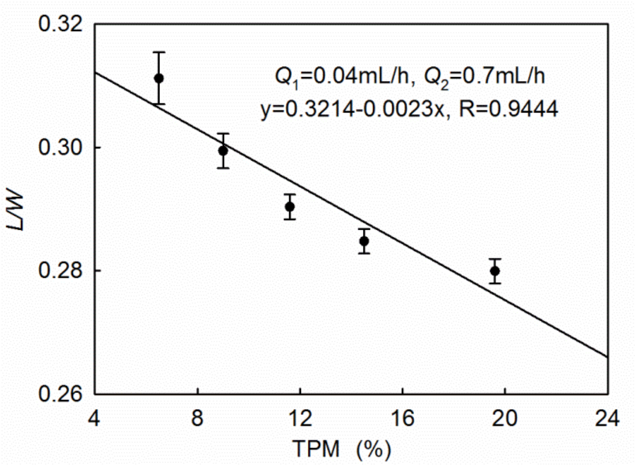

Supplement Fig. 3: Correlation between experimentally measured L/W and experimentally measured TPM. The relationship is highly linear and proves that L/W is a suitable parameter for discriminating oil samples FO0-FO4. W=1000µm, h=40 µm.

Supplement Fig. 3 confirms that normalized steady-state length (L/W) linearly correlates to TPM. The sensitivity under flow rates of Q_1=0.04mL/h and Q_2=0.7mL/h was determined to be 0.0023/TPM, and the precision to be ±1.13% TPM. The oil should be replaced when the steady-state length of the droplets drops to around 266.2µm (corresponding to TPM of 24%) under these flow rates.

3. Experimental validation of model

Eq. (1) in the paper can be written as

$$ln\left(\frac{L}{W}\right) = ln(m) + nln(\alpha_2 Ca_2)$$

This relationship is confirmed experimentally, as shown in Supplement Fig. 4 where experimental data collected under different Q_1 and Q_2 flow rates form lines having approximately equal slopes.

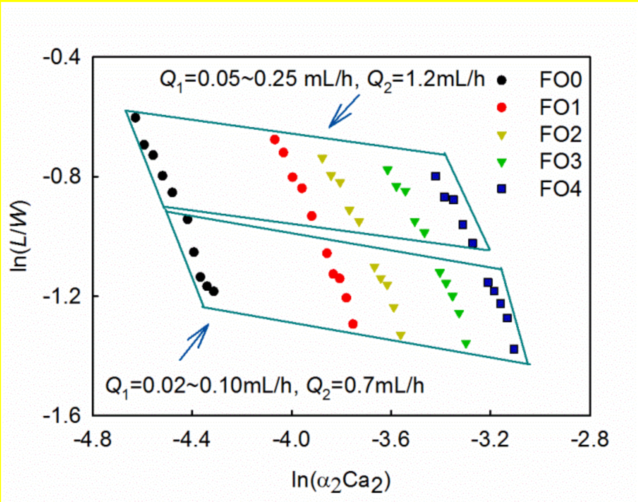

Supplement Fig. 4: The relationship between ln(*L*/*W*) and ln($\alpha_2 Ca_2$). It can be seen in the figure that data collected under different flow rates form lines of approximately equal slopes. Microchannel width *W*=1000 μm, microchannel height *h*=80 μm (upper data), *h*=40 μm (lower data).

4. Effects of viscosity and interfacial tension

The changes of *L*/*W* caused by viscosity differences is

$$\left(\Delta\frac{L}{W}\right)_{\eta_2} = \frac{\partial(\frac{L}{W})}{\partial\eta_2}\Delta\eta_2 = mn(\alpha_2 Ca_2)^n \frac{\Delta\eta_2}{\eta_2}$$

and the changes of *L*/*W* caused by interfacial tension differences is

$$\left(\Delta\frac{L}{W}\right)_{\gamma_{12}} = \frac{\partial(\frac{L}{W})}{\partial\gamma_{12}}\Delta\gamma_{12} = -mn(\alpha_2 Ca_2)^n \frac{\Delta\gamma_{12}}{\gamma_{12}}$$

From the above two equations it can be seen that $\frac{\Delta\eta_2}{\eta_2}$ and $\frac{\Delta\gamma_{12}}{\gamma_{12}}$ have coefficients of the same magnitude, i.e., $mn(\alpha_2 Ca_2)^n$. This identical magnitude indicates that changes of viscosity and interfacial tension both affect *L*/*W* changes, and neither dominates the other.
